# Primary bladder mucosa-associated lymphoid tissue lymphoma: A case report and literature review

**DOI:** 10.1097/MD.0000000000020825

**Published:** 2020-07-10

**Authors:** Hewei Xu, Zhengsen Chen, Baixin Shen, Zhongqing Wei

**Affiliations:** Urology Department, The Second Affiliated Hospital of Nanjing Medical University, Nanjing, Jiangsu, China.

**Keywords:** bladder, case report, mucosa-associated lymphoid tissue, transurethral resection of bladder tumors

## Abstract

**Introduction:**

Primary bladder mucosa-associated lymphoid tissue (MALT) lymphoma is a rare tumor. To date, the PubMed database contains only 39 English articles covering 63 cases of primary bladder MALT lymphoma. Herein, we report a case of this disease and review the current literature.

**Patient concerns:**

A 77-year-old woman presented with frequent urination, urinary urgency, and dysuria for 3 years. In the past 3 years, the patient's symptoms recurred and progressively worsened, and she was admitted to the hospital.

**Diagnosis:**

A histopathological examination revealed the bladder mass as a tumor with high proliferation of atypical B-lymphocytes. Immunohistochemistry showed positive results for CD20, PAX-5, Ki-67, *BCL-2*, and CD21 and negative results for CD10, MUM1, TDT, and cyclin D1. These data supported the diagnosis of primary bladder MALT lymphoma.

**Interventions:**

A transurethral resection of bladder tumor was performed to treat the disease.

**Outcomes:**

The patient was alive and healthy at the 15-month follow-up.

**Conclusion:**

Primary bladder MALT lymphoma is a rare disease and can be easily missed or misdiagnosed before achieving a histological confirmation. Surgery may be the best choice for both diagnosis and treatment.

## Abstract 2

Supplemental Digital Content is available in the text

## 1 Introduction

Mucosa-associated lymphoid tissue (MALT) is a unique subtype of B-cell non-Hodgkin's lymphoma, and is a low-grade malignant tumor.^[1]^ Primary MALT lymphoma of the bladder is rare. To date, the PubMed database contains only 39 English articles covering 63 cases of primary bladder MALT lymphoma, of which only 38 include description on clinical manifestations, treatments, and outcomes.^[2–40]^ The cause of this disease is unknown, and there is no consensus on treatment approach. Herein, we report and discuss a case of primary bladder MALT lymphoma and its treatment. We also analyze the clinical characteristics, diagnosis, and treatment of this disease by reviewing the current literature.

## 2 Case report

**Figure 1:**
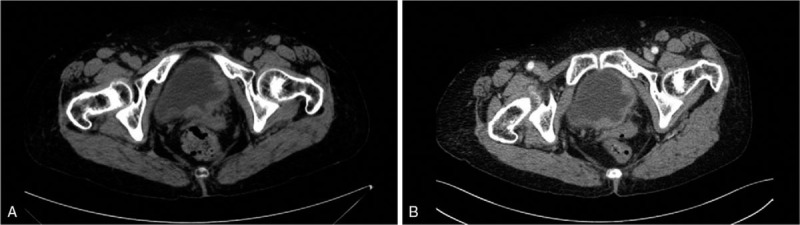
(A-B): Pelvic contrast-enhanced computed tomography (CT) showed multiple nodules in the bladder wall.

**Figure 2:**
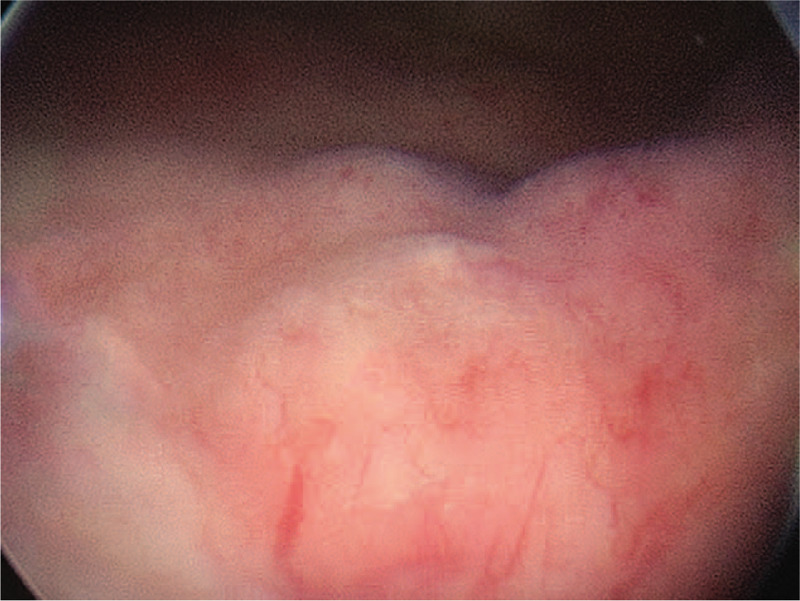
Cystoscopy showed “bladder occupancy”.

A 77-year-old woman presented with frequent urination, urinary urgency, and dysuria for 3 years. She had pain after urination, without gross hematuria, and the symptoms had improved after anti-inflammatory treatment. In the past 3 years, the patient's symptoms recurred and progressively worsened, and she was admitted to the hospital on December 1, 2017. She reported no history of smoking, hepatitis, tuberculosis, connective tissue disease, drug or food allergies, or chronic cystitis. The superficial lymph nodes were not tender or swollen, and there was no ulcer in the mouth. No abnormalities were detected in the heart or abdomen. B-scan ultrasonography of the urinary system showed roughness in the inner walls of the bladder, and pelvic contrast-enhanced computed tomography (CT) showed multiple nodules in the bladder wall (Fig. 1A, 1B). CT of the chest and abdomen showed no swelling on lymph nodes. Bone marrow biopsy, gastrointestinal endoscopy, and abdominal color Doppler ultrasonography showed no abnormalities. Further cystoscopy improved the relevant findings by showing a “bladder occupying” tumor (Fig. 2).

**Figure 3:**
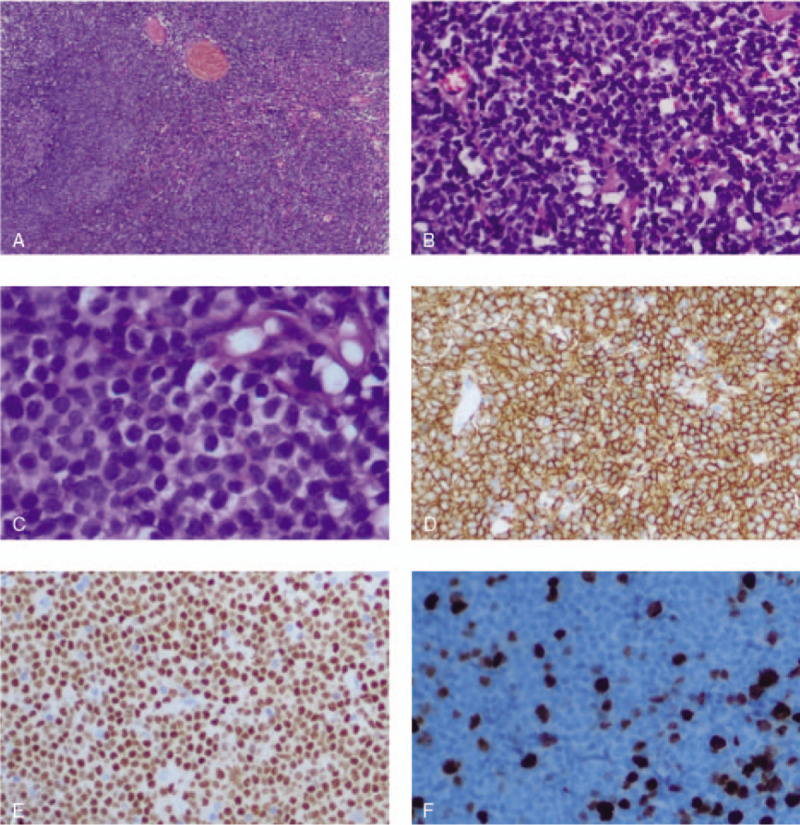
Characteristics of histological examination. The lesion consisted of dense lymphocyte infiltration with some lymphoid follicles (A, Hematoxylin and Eosin, HE 40x magnification). Small-to middle-sized, centrocyte-like lymphoid cell population were diffusely infiltrated with lymphoepithelial lesions of bladder (B, HE 200x magnification and C, HE 400x magnification). And lymphocytes were diffusely positive for CD20 (D 200x magnification) and pax-5 (E 200x magnification) antibodies. Ki67 labeling index was in a low level (F 200x magnification).

Histopathology of the bladder tumor revealed a “bladder occupying”, high proliferation of lymphatic tissue. Immunohistochemistry showed positive results for CD20, PAX-5, Ki-67, *BCL-2*, and CD21, but negative results for CD10, MUM1, TDT, and cyclin D1. The pathological diagnosis of the “bladder occupying” tumor was consistent with MALT lymphoma (Fig. 3).

The patient underwent cystoscopy and transurethral resection of bladder tumors (TURBT) under general anesthesia using a laryngeal mask to excise the tumor. The clinical stage IE group A was confirmed based on the clinical and imaging examinations. According to the patient's condition, the patient was recommended radiotherapy and chemotherapy, which was rejected by the patient and family. Therefore, regular follow-up was performed. At the March 2019 follow-up, there were no abnormalities in the chest and abdominal CT, which was similar to the previous findings. Simultaneously, fluorodeoxyglucose-positron emission tomography/CT (FDG-PET/CT) showed no lesions other than those in the bladder.

## 3 Discussion

MALT lymphoma is a non-Hodgkin's lymphoma that causes B-cell proliferation in the mucosal margin. It is characterized by inert growth and a tendency to be localized in the primary area for a long time.^[41]^ The concept of MALT lymphoma was first proposed by Isaacson and Wright in 1983.^[42]^ MALT lymphoma invades many mucosal organs, of which stomach is the most common, followed by the lungs, while primary bladder MALT lymphoma is rare, accounting for approximately 0.2% of non-Hodgkin's lymphoma cases.^[29,43,44]^ Kuhara et al^[45]^ reported the first case of bladder MALT lymphoma in a woman in 1990. To date, the published literature on bladder MALT lymphoma consists mostly of case reports.

The clinical features and treatment of bladder MALT lymphoma are unclear. Table 1 summarizes the clinical features of the reported cases in a systematic review of the bladder MALT lymphoma, including the present case. We found that the average age of onset of the disease was 65 years (17–88 years). Women were more commonly affected, with the men/women ratio of about 1:5, which was consistent with previous literature.^[34,46,47]^ The first symptom of the patient was mostly hematuria (50.9%). Most patients (76.5%) presented with a solitary mass. According to the Ann Arbor staging system, most patients (89.5%) belonged to stage IE.

**Table 1:**
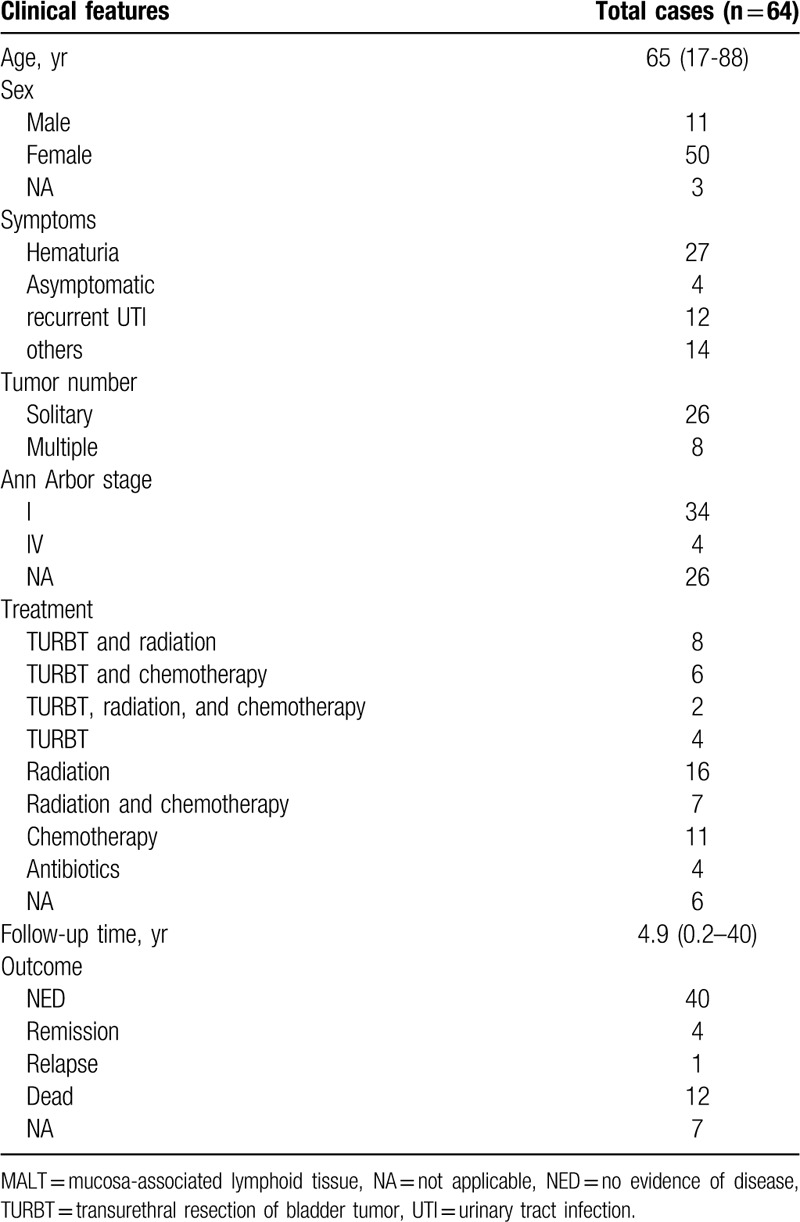
Clinical features of the reported cases of primary bladder MALT lymphoma, including the present case.

The pathogenesis of primary bladder MALT lymphoma is unknown. At present, it is believed that bladder MALT lymphoma is caused by chronic inflammation. Chronic inflammation leads to the acquisition of MALT in the bladder mucosa, which progresses to lymphoma. Similar to the association between *Helicobacter pylori* and gastric MALT, Oscier et al reported that *E coli* is a common infectious agent for the pathogenesis of bladder MALT lymphoma, and patients can achieve complete remission after anti-infective treatment.^[48]^ Bladder MALT lymphoma has also been reported in autoimmune interstitial cystitis, which is similar to the association between thyroid MALT lymphoma and chronic lymphocytic thyroiditis.^[27]^ We analyzed the cases of a total of 64 patients with bladder MALT lymphoma, including the present case, of which 12 had recurrent urinary tract infection and 1 had interstitial cystitis. Four patients were treated with antibiotics; of them, 1 showed marked reduction in tumor size, and 3 had “no evidence of disease”. These results indicate that chronic inflammation and autoimmune disease may contribute to the development of primary bladder MALT lymphoma. However, further evidence is needed to reach a conclusion.

Most cases of primary bladder MALT lymphoma have a long disease course, insidious onset, and non-specific clinical manifestations. The most common clinical manifestation is gross hematuria, followed by dysuria, nocturia, frequent urination, urinary urgency, and painful urination. A few patients have complained of perineal pain and urinary incontinence. Only a few patients could locate the lower abdomen mass at presentation. Patients with this disease often receive delayed diagnosis and treatment due to the lack of specific symptoms and signs. The clinical manifestations in the present case were frequent urination, urinary urgency, and dysuria; thus, the diagnosis could not be made without pathological and immunohistochemical examinations. Typical MALT lymphoma cells often present as small to medium-sized lymphocytes with moderate cell mass and nuclear irregularities, similar to the follicular center cells, and are thus called “central cell-like cells.” In immunohistochemistry, MALT lymphoma is positive for CD20 and CD79a and negative for CD5, CD10, CD23, and cyclin D1. In addition, lymphoepithelial lesions, colonization of reactive follicles by monocytoid cells, and plasmacytic differentiation were observed in the transitional epithelium.^[8,24]^ In this study, the clinical symptoms, pathological histomorphology, and immunophenotype of the patients were found to be consistent with MALT lymphoma.

To date, an optimal treatment strategy for primary bladder MALT lymphoma has not been determined because of the rarity of the disease. Of all the cases in this study, 89.5% of patients present of localized (stage I) disease. A wide variety of methods were treated and most cases had positive outcomes, with a mean follow-up duration of 4.9 years (range: 0.2–40 years) (Table 1). Combined with literature reports, we recommend that surgery like TURBT be tried first, followed by chemotherapy, or radiotherapy alone, or combination therapy. Radiotherapy alone proved effective in only 68.8% (11 of 16) patients. As for the chemotherapy regimen, rituximab (anti-CD20 monoclonal antibody) has been shown to be useful for MALT lymphoma, with a remission rate of 55% to 73%.^[49]^ In the present case, the patient just underwent TURBT and She has remained disease-free without any additional treatment after the surgery (15-month follow-up). However, by accumulating more cases and long-term follow-up, the best treatment and prognosis of the disease can be better understood.

## 4 Conclusion

In summary, primary bladder MALT lymphoma is a rare tumor, which tends to be a solitary mass, most commonly occurring in the elderly women. Because of the lack of specific clinical symptoms, signs, and imaging findings, it is easily missed or misdiagnosed before achieving a histological confirmation. Surgery may be the best choice for both diagnosis and treatment. Primary bladder MALT lymphoma is an indolent tumor with a good overall prognosis. We need additional reports to improve our understanding of the disease and its management. Supplementary Table.

## Author contributions

Dr. Baixin Shen and Dr. Zhongqing Wei contributed equally to the work.

**Conceptualization:** Zhongqing Wei, Baixin Shen.

**Data curation:** Zhongqing Wei, Baixin Shen.

**Formal analysis:** Hewei Xu.

**Funding acquisition:** Zhongqing Wei, Baixin Shen.

**Investigation:** Hewei Xu.

**Methodology:** Hewei Xu.

**Software:** Zhengsen Chen.

**Supervision:** Zhongqing Wei, Baixin Shen.

**Writing – original draft:** Hewei Xu.

## Supplementary Material

Supplemental Digital Content
