# Supplementary material for: Primary bladder mucosa-associated lymphoid tissue lymphoma: A case report and literature review
Source: Medicine (Baltimore). 2020 Jul 10;99(28):e20825. doi: 10.1097/MD.0000000000020825 (PMC7360252; doi:10.1097/MD.0000000000020825)
Supplement: Supplemental Digital Content [file medi-99-e20825-s001.docx]

**Supplementary Table**

| **Ann Arbor stage** | **Treatment** | **Total cases** | **Outcome** |
| --- | --- | --- | --- |
| I | TURBT and Radiation | 2 | NED |
|  | TURBT and Chemotherapy | 6 | NED or Dead (1) |
|  | TURBT, Radiation and Chemotherapy | 1 | Remission |
|  | TURBT | 2 | NED or Remission (1) |
|  | Radiation | 6 | NED |
|  | Radiation and Chemotherapy | 4 | NED |
|  | Chemotherapy | 5 | NED |
|  | Antibiotics | 3 | NED or Remission (1) |
|  | NA | 5 | NA |
| IV | TURBT and Radiation | 1 | Remission |
|  | Radiation | 1 | NED |
|  | Radiation and Chemotherap | 1 | Dead |
|  | Chemotherapy | 1 | NED |
| NA | TURBT and Radiation | 5 | NED or Dead (1) |
|  | TURBT, Radiation and Chemotherapy | 1 | NED |
|  | TURBT | 2 | NED |
|  | Radiation | 9 | NED or Dead (5) |
|  | Radiation and Chemotherapy | 2 | NED or Dead (1) |
|  | Chemotherapy | 5 | NED or Dead (2) |
|  | Antibiotics | 1 | Recurrence Stomach |
|  | Untreated | 1 | Dead |

**Clinical information of different stages of treatment and prognosis of primary bladder MALT lymphoma, including the present case.**

MALT = mucosa-associated lymphoid tissue, NA = not applicable, TURBT = transurethral resection of bladder tumor, NED = no evidence of disease.
